# Estimating the probability of stroke in Korean hypertensive patients visiting tertiary hospitals using a risk profile from the framingham study

**DOI:** 10.1186/1471-2377-9-16

**Published:** 2009-04-22

**Authors:** Cheol Ung Choi, Chang Gyu Park

**Affiliations:** 1 Cardiovascular Center, Korea University Guro Hospital, Seoul, Korea

## Abstract

**Background:**

Hypertension is the most important single modifiable risk factor for stroke. We investigated the distribution of stroke risk factors and 10-year probability of stroke in Korean hypertensive patients.

**Methods:**

A total of 1,402 hypertensive patients treated by cardiology departments at 37 general hospitals nationwide were enrolled. Risk factors for stroke were evaluated using a series of laboratory tests and physical examinations, and the 10-year probability of stroke was determined by applying the Framingham stroke risk equation.

**Results:**

The proportion of patients who have uncontrolled hypertension despite use of antihypertensives was 37.2% (37.2% women, 37.3% men, p = 0.990). The average 10-year probability of stroke in hypertensive patients was 24.27% (24.17% women, 24.39% men, p = 0.825), approximately 2.4 times higher than of the risk of stroke observed in the Korean Cancer Prevention Study [KCPS] cohort. The 10-year stroke probability in patients with hypertension increased in proportion to age. In patients for hypertension, the 10-year probability of stroke increased in proportion to blood pressure.

**Conclusion:**

Aggressive interventions are mandated to reduce blood pressure and alleviate the high risk of stroke in hypertensive patients.

## Background

Hypertension (HTN) is one of the most common chronic diseases in adults. Major complications of HTN include ischemic heart disease, stroke, heart failure, renal failure, aortic and peripheral arterial diseases. Stroke is a leading cause of death and disability among individuals older than 65 years in Korea. The total number of deaths attributable to stroke in Korea is estimated at 13.9% and associated annual medical costs account for more than 10% of total medical expenditures [1,2]. In addition, stroke is becoming a major cause of death and behavioral disorders due to the rapidly aging Korean population [3].

Although various factors induce stroke, HTN and aging are known to be most common risk factors of stroke worldwide [4,5]. Aging is an irreversible factor, but HTN is controllable. Appropriate treatment of HTN has been shown to reduce the risk of stroke by 40% and the risk of acute myocardial infarction by 15% [6]. According to related research, an increase of systolic blood pressure (SBP) and diastolic blood pressure (DBP) 20/10 mmHg from 115/75 mmHg doubles the risk of stroke [6].

The Framingham Study, a reliable prospective epidemiology study on chronic diseases established in the United States in 1948, revealed that risk factors of stroke include age, SBP, use of anti-hypertensives, diabetes, smoking, cardiovascular diseases (CVD), atrial fibrillation, and left ventricular hypertrophy (LVH) [7-9]. The average 10-year probability of stroke in the total Korean Cancer Prevention Study (KCPS) cohort was 3.5% for males and 3.7% for females, and the average 10-year probability of stroke in the 55–84 years of age group within the KCPS cohort was 10.0% for males and 9.0% for females [10]. Wolf *et al*. showed that for each 10 mmHg increase in SBP, the risk of stroke increased by 91% for men and 68% for women [7]. Although hypertension is the most important single modifiable risk factor for stroke, there is little data about the practical 10 year stroke risk in hypertensive patients. We reported previously that the 10-year risk of stroke in Korean hypertensive patients visiting community-based hospitals was approximately 4.6 times higher than of the risk of stroke in of the KCPS cohort [11]. However, the enrolled patients were limited to only patients visiting community-based hospitals. Therefore, we planned to evaluate the 10-year probability of stroke in Korean hypertensive patients visiting tertiary hospitals using the Framingham risk score [7].

## Methods

### Study population

We conducted a multicenter study evaluating patients treated by the cardiology departments of 37 general hospitals in Korea. Our study population included 1735 patients with HTN between 55 and 84 years of age. We excluded 333 subjects due to a previous history of stroke. Consequently, 1402 patients (769 male, 633 female) were enrolled in the study. All subjects gave written informed consent. This study was approved by the local ethics committee.

### Laboratory and lifestyle factors measurement

Body weight and height were measured with subjects wearing light clothing and without shoes. The body mass index (BMI) was calculated as the weight in kilograms divided by the height in meters squared. The waist circumference was measured from the narrowest point between the lower borders of the rib cage and the iliac crest.

Blood pressure was measured in the right arm, using an appropriately sized cuff and a standard mercury sphygmomanometer, after the subjects had been seated for at least 15 min, with feet on the floor and arm supported at heart level.

Fasting blood samples were obtained in the morning after at least 8 hours of fasting. Plasma glucose, total cholesterol, triglyceride, high density lipoprotein (HDL)-cholesterol, and low density lipoprotein (LDL)-cholesterol were measured.

Risk factors for stroke were age, diabetes, smoking, medical history of cardiovascular disease, atrial fibrillation, LVH, hyperlipidemia, alcohol intake, physical exercise, obesity, and family history of stroke.

Antihypertensive medications that subjects were currently receiving were classified as angiotensin converting enzyme inhibitor (ACEI), angiotensin receptor blocker (ARB), calcium channel blocker (CCB), beta-blocker (BB), diuretics, and others.

### Estimation of 10-year probability of stroke

The 10-year probability of stroke was estimated for each patient using the Framingham risk score [7]. The variables used to calculate the Framingham risk score are age, systolic blood pressure, anti-hypertensive therapy, diabetes mellitus, smoking, CVD, atrial fibrillation, and LVH.

### Study definitions

We identified HTN with repeated measurements of ≥ 140 mmHg SBP or ≥ 90 mmHg DBP or previous diagnosis. HTN stage was classified according to Joint National Committee 7 (JNC-7) criteria [12]: normal (SBP < 120 mm Hg and DBP < 80 mm Hg); pre-HTN (120 ≤ SBP < 140 mm Hg or 80 ≤ DBP < 90 mm Hg); stage 1 (140 ≤ SBP < 160 mm Hg or 90 ≤ DBP < 100 mm Hg); and stage 2 (SBP ≥ 160 or DBP ≥ 100 mm Hg). Uncontrolled HTN was defined as repeated measurements of ≥ 140 mmHg SBP and ≥ 90 mmHg DBP, despite use of antihypertensives. Diabetes was defined as fasting blood glucose concentration ≥ 126 mg/dL or taking diabetes medications. Subjects were classified as "non-smokers" (patients who never smoked before and did not smoke currently) or "smokers" (patients who had smoked within the past year or who smoke currently). Atrial fibrillation was confirmed by electrocardiogram. The product of QRS duration times the Cornell voltage combination (R_aVL _+ S_V3_, with 6 mm added in women [13,14]) greater than 2440 mm/msec or Sokolow-Lyon voltage (S_V1 _+ RV_5/6_) greater than 38 mm [15] was used to identify LVH.

CVD was defined as a combination of coronary artery disease (CAD) and congestive heart failure (CHF). CAD was defined as any hospitalization for acute myocardial infarction or angina and CHF was defined as any hospitalization for CHF. If a stroke had occured within a first line relative, subjects were considered to have a family history of stroke. Dyslipidemia was defined in terms of the following criteria: total cholesterol > 200 mg/dL, triglyceride > 150 mg/dL, HDL-cholesterol < 40 mg/dL, or LDL-cholesterol > 100 mg/dL.

We use the definition of metabolic syndrome as proposed by National Cholesterol Education Program Adult Treatment Panel III [16]. Target organ damage variables were serum creatinine (women > 1.2 mg/dL, men > 1.3 mg/dL), LVH (electrocardiography criteria per Cornell, and/or Sokolow-Lyon criteria [13,15]), microalbuminuria (women ≥ 3.5 mg/mmol in women, men ≥ 2.5 mg/mmol per 2003 guidelines of the European Society of Hypertension/European Society of Cardiology) [17], and retinal lesions following Dodson's classification [18]. Renal function alteration was also calculated, using the Cockcroft and Levey formulae, and expressed as a glomerular filtrate < 60 ml/min.

### Statistical analysis

Statistical analysis was performed using the SPSS 10.0 software package (SPSS Inc., Chicago, IL, USA). Continuous variables were expressed as means ± S.D. and categorical variables were reported as number (%). Continuous variables were compared using Student's t-test, or one way-ANOVA with *post hoc *multiple comparisons performed using Duncan's (D) multiple comparison test. Categorical variables were compared using a chi-square test or Fisher's exact test. A p < 0.05 was considered statistically significant.

## Results

### Baseline characteristics and risk factors

**Figure 1:**
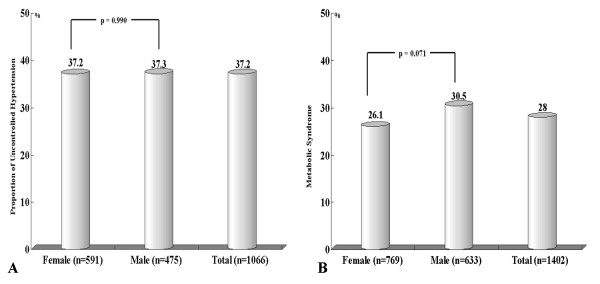
**A: Proportion of patients with uncontrolled hypertension despite use of antihypertensives**. Uncontrolled HTN was defined as repeated measurements of ≥ 140 mmHg SBP and ≥ 90 mmHg DBP, despite use of antihypertensives. B: Prevalence of metabolic syndrome in patients with hypertension.

The baseline characteristics and risk factors of our subjects are summarized in Table 1. Our sample included 633 men and 769 women. The mean age of patients was 68.41 ± 7.40 years. The average SBP was 132 ± 17 mmHg and the average DBP was 79 ± 10 mmHg. The prevalence of subjects taken antihypertensives was 76.0% (76.9% women, 75.0% men, p = 0.429). The prevalence of subjects with target organ damage was 16.3% (15.2% women, 17.5% men, p = 0.241). The prevalence of diabetes mellitus was 16% (17.2% women, 14.7% men, p = 0.209) and the prevalence of cigarette smoking was 30.6% (3.6% women, 63.3% men, p < 0.001). The prevalence of CVD was 49.5% (47.5% women, 52.0% men, p = 0.093) and the prevalence of atrial fibrillation was 7.7% (7.0% women, 8.5% men, p = 0.292). The prevalence of LVH was 32.2% (28.3% women, 36.8% men, p < 0.001). Measurements of total cholesterol, HDL-cholesterol and LDL-cholesterol measurements were higher in women than in men. Prevalences of smoking, LVH, drinking, and physical exercise were higher in men than in women. The proportion of patients who had uncontrolled hypertension despite use of antihypertensives was 37.2% (37.2% women, 37.3% men, p = 0.990; Figure. 1-A) The prevalence of metabolic syndrome according to the ATP-III guideline was 28% (26.1% women, 30.5% men, p = 0.071; Figure 1-B).

**Table 1:** Characteristics of risk factors in study subjects

	Female	Male	Total	
		
Risk factors	(n = 769)	(n = 633)	(n = 1402)	p
Age, years	68.96 ± 7.36	67.74 ± 7.40	68.41 ± 7.40	0.002
Height,	154.50 ± 6.20	167.24 ± 5.79	160.44 ± 8.76	<0.001
Weight	60.63 ± 8.68	69.08 ± 9.23	64.56 ± 9.88	<0.001
Systolic blood pressure, mmHg	133 ± 17	131 ± 16	132 ± 17	0.143
Diastolic blood pressure, mmHg	79 ± 10	79 ± 10	79 ± 10	0.303
Heart rate	71 ± 11	71 ± 11	71 ± 11	0.648
Glucose	115.12 ± 34.14	113.62 ± 37.13	114.43 ± 377	0.491
Total cholesterol	201.32 ± 42.02	184.42 ± 41.03	193.56 ± 42.39	< 0.001
HDLc	49.52 ± 29.82	44.66 ± 12.43	47.28 ± 23.56	< 0.001
Triglyceride	159.85 ± 127.34	156.05 ± 108.74	158.08 ± 118.99	0.573
LDLc	132.00 ± 42.22	115.20 ± 42.10	12434 ± 42.96	< 0.001
Antihypertensives, n (%)	591 (76.9)	475 (75.0)	1066 (76.0)	0.429
Target organ damage, n (%)	117 (15.2)	111 (17.5)	228 (16.3)	0.241
Diabetes mellitus, n (%)	132 (17.2)	93 (14.7)	225 (16)	0.209
Diabetes mellitus medication, n (%)	152 (19.8)	103 (16.3)	255 (18.2)	0.091
Cigarette smoking, n (%)	28 (3.6)	401 (63.3)	429 (30.6)	< 0.001
Cardiovascular disease, n (%)	365 (47.5)	329 (52.0)	694 (49.5)	0.093
Atrial fibrillation, n (%)	54 (7.0)	54 (8.5)	108 (7.7)	0.292
Left ventricular hypertrophy, n (%)	218 (28.3)	233 (36.8)	451 (32.2)	0.001
Dyslipidemia, n (%)	116 (16.6)	47 (7.9)	163 (12.6)	< 0.001
Body mass index ≥ 25 kg/m^2^, n (%)	231 (30)	149 (23.5)	380 (27.1)	0.006
Stroke family history, n (%)	94 (12.2)	95 (15.0)	189 (13.5)	0.129
Antiplatelet therapy, n (%)	367 (47.7)	386 (61.0)	753 (53.7)	< 0.001
Warfarin therapy, n (%)	22 (2.9)	17 (2.7)	39 (2.8)	0.743
Lipid lowering therapy, n (%)	238 (30.9)	167 (26.4)	405 (28.9)	0.06
Uncontrolled HTN, n (%)	277 (36)	229 (36.2)	506 (36.1)	0.736
10-year probability of stroke	24.17 ± 20.77	24.39 ± 17.03	24.27 ± 19.16	0.825

HDLc: High density lipoprotein-cholesterol, LDLc: Low density lipoprotein-cholesterol, cardiovascular disease: history of myocardial infarction, angina pectoris, coronary insufficiency, intermittent claudication, or congestive heart failure. Values: Mean ± SD or number (%). n: number.

### Average 10-year probability of stroke

**Figure 2:**
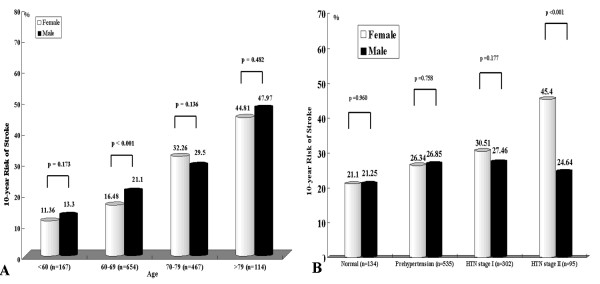
**A: The average 10-year risk of stroke according to age in men and women with hypertension**. B: The difference in average 10-year probability of stroke between men and women, according to HTN stage. Normal is SBP < 120 mmHg and DBP < 80 mmHg, Pre-hypertension is 120 ≤ SBP < 140 mmHg or 80 ≤ DBP < 90 mmHg, HTN stage 1 is 140 ≤ SBP < 160 mmHg or 90 ≤ DBP < 100 mmHg, HTN stage 2 is SBP ≥ 160 or DBP ≥ 100 mmHg.

The average 10-year probability of stroke was 26.27% (26.9% women, 25.5% men) (Table 1). As expected, 10-year probability of stroke in patients with HTN increased in proportion to age (Table 2). Figure 2-A illustrates differences in the average 10-year probability of stroke between men and women according to age. Men aged 60–69 years had a higher 10-year probability of stroke than women of similar age. However, in other age groups, there were no gender-related differences. Table 2 also showed the average 10-year probability of stroke according to blood pressure in treated hypertensive patients. Differences in the average 10-year probability of stroke between men and women, according to HTN stage, are shown in Figure 2-B. In HTN stage 2, the 10-year probability of stroke was high in women compared to men. At all other HTN stages there were no differences by gender.

**Figure 3:**
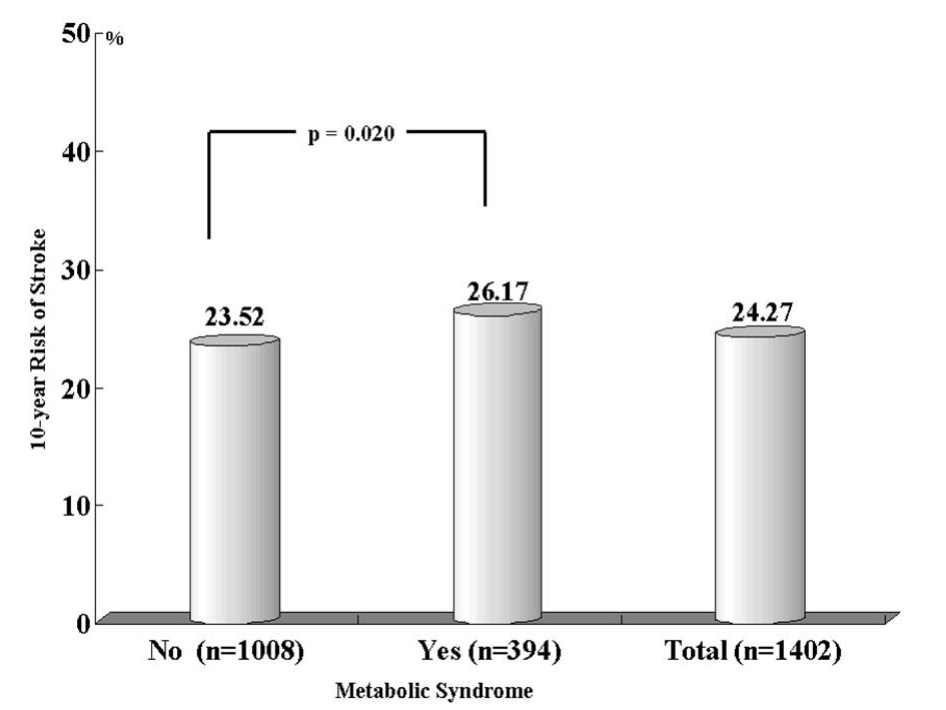
**The average 10-year risk of stroke according to metabolic syndrome**.

As illustrated in Figure 3, the average 10-year probability of stroke was higher in patients with metabolic syndrome than in patients without metabolic syndrome.

**Table 2:** Average 10-year probability of stroke according to age in all subjects and blood pressure in treated hypertensive patients.

Age group	10 year risk of Stroke*	Stage of HTN	10 year risk of Stroke*
< 60 (n = 167)	12.42 ± 9.11^a^	Normal (n = 134)	21.16 ± 17.23^a^
60–69 (n = 654)	18.63 ± 13.89^b^	Prehypertension (n = 535)	26.57 ± 18.92^b^
70–79 (n = 467)	31.10 ± 20.42^c^	Stage 1 HTN (302)	29.15 ± 19.48^b^
> 79 (n = 114)	46.00 ± 23.15^d^	Stage 2 HTN (95)	36.22 ± 23.74^c^
P**	< 0.001	P**	<0.001

* Mean ± standard deviation

**P-value is for one-way analysis of variance

^a,b,c^, same letters indicate statistical insignificance based on Duncan's multiple comparisons. Normal: SBP < 120 mm Hg and DBP < 80 mm Hg, pre-hypertension: 120 ≤ SBP < 140 mm Hg or 80 ≤ DBP < 90 mm Hg, stage 1: 140 ≤ SBP < 160 mm Hg or 90 ≤ DBP < 100 mm Hg, stage 2: SBP ≥ 160 or DBP ≥ 100 mm Hg.

### Concomitant antihypertensives taken according to age (Table 3)

**Table 3:** Concomitant antihypertensives according to age

**Drug, n (%)**	**< 60**	**60–69**	**70–79**	**> 79**	**Total**	
		
	(n = 167)	(n = 654)	(n = 467)	(n = 114)	(n = 1402)	p
ACEI	25 (15.0)	53 (8.1)	48 (10.3)	13 (11.4)	139 (9.9)	0.057
ARB	76 (45.5)	325 (49.7)	220 (47.1)	60 (52.6)	681(48.6)	0.55
BB	49 (29.3)	203 (31.0)	156 (33.4)	34 (29.8)	442 (31.5)	0.716
CCB	66 (39.5)	261 (39.9)	176 (37.7)	39 (34.2)	542 (38.7)	0.655
Diuretics	9 (5.4)	39 (6.0)	52 (11.1)	10 (8.8)	110 (7.8)	0.008
Combined Antihypertensives	55 (32.9)	222 (33.9)	183 (39.2)	43 (37.7)	503 (35.9)	0.253

ACEI: angiotensin converting enzyme inhibitor, ARB: angiotensin receptor blocker, BB: beta-blocker, CCB: calcium channel blocker. Combined antihypertensive treatment: take more than two antihypertensive drugs.

Physicians prescribed antihypertensives for hypertensive patients was as follows: ARB 48.6%, CCB 38.7%, beta-blocker 31.5%, ACEI 9.9%, diuretics 7.8%, combined treatment 35.9%. Of these, only diuretics were prescribed more frequently with increasing age, with the rest of these medications showing no age-related presciption trends.

## Discussion

This study is an initial report designed to predict the 10-year probability of stroke in Korean hypertensive patients visiting tertiary hospitals using the Framingham risk score.

We found the average 10-year probability of stroke in hypertensive patients was 26.27% (26.9% women, 25.5% men), which was approximately 2.4 times higher than values found in a previous study of the KCPS cohort [10]. This result may be explained by the fact that 34% of the hypertensive patients did not take antihypertensives (Table 1) and therefore their blood pressure was not well controlled. Another possible explanation is that even among hypertensive patients complying with treatment, 37.2% suffered from uncontrolled HTN (Figure 1-A). The 10-year probability of stroke increased in proportion to blood pressure even among patients being treated for hypertension (Table 2). These findings demonstrate that stricter control of HTN may be an important intervention for prevention of stroke.

In HTN stage 2, the 10-year probability of stroke was higher in women compared to that of men, but in other HTN stages, there was no difference between men and women (Figure 2-B). Female patients in HTN stage 2, were older than their male counterparts, but this difference was not observed in other HTN stages. In addition, the distributions of other risk factors did not segregate by gender at any other stage of HTN (data not shown). Therefore, we believe that the difference in the 10-year probability of stroke between men and women for HTN stage 2 is related to age.

The 10-year probability of stroke in HTN patients increased in proportion to age (Table 2) but the distribution of 10-year probability of stroke between men and women varied according to age. In the 60–69 year age group, the 10-year probability of stroke was higher for men than for women, but in other age groups there was no difference by gender (Figure 2-A). In the 60–69 year age group, frequencies of history of CVD and LVH were higher for men than for women, but not in other age groups. The distribution of other risk factors between men and women did not differ according to age (data not shown). Therefore, we believe that differences in the 10-year probability of stroke between men and women in aged 60–69 are driven by the distribution of CVD and LVH.

The prevalence of diabetes mellitus in hypertensive patients was 16% (17.2% women, 14.7% men; Table 1), which was higher than that seen in the Korean National Health and Nutrition Examination Survey [19]. This study showed that, in the Korean population, the prevalence of diabetes mellitus was 9.1% (7.9% women, 10.2% men). Our enrolled subjects were older and considered high risk patients, which may explain the discrepancy.

The prevalence of dyslipidemia in our sample of hypertensive patients was 12.6% (7.9% women, 16.6% men; Table 1). This prevalence was lower than that observed by the Korean National Health and Nutrition Examination Survey (2005) [19]. That study showed that, in the general Korean population, the prevalence of dyslipidemia was 48.3% (38.2% women, 58.9% men). Our enrolled subjects were older and considered high risk patients, and had been prescribed anti-lipid lowering agents, which may explain the discrepancy.

The prevalence of metabolic syndrome in our sample of hypertensive patients measured according to the ATP-III guideline was 28% (26.1% women, 30.5% men; Figure 1-B). This result was similar to values found by the Korean National Health and Nutrition Examination Survey (2005) [19]. That study showed that, in the general Korean population, the prevalence of metabolic syndrome was 29.5% (26.1% women, 33.1% men). Another report suggests that metabolic syndrome is an important risk factor for ischemic stroke [20]. According to our results, the 10-year risk of stroke is higher for patients suffering from metabolic syndrome (Figure 3).

In Korea, ARB was the most frequently prescribed antihypertensive at 48.6%, with the next being CCB (38.7%). Cardiologists working at general hospitals tend to prefer ARB to other antihypertensives due to higher compliance and fewer side effects., Many clinical papers demonstrating positive effects of ARB for the prevention of stroke and cardiovascular diseases have been published recently [21]. The prescription frequency of ACEI was only 9.9%, probably due to side effects such as cough, and a general preference for ARB. Diuretics were more frequently prescribed for older patients than for younger patients. This may be due to a high prevalence of complications, such as ischemic heart disease and heart failure, in older patients. Among Korean hypertensive patients, 35.9% took more than two antihypertensive drugs (Table 3). This frequency was similar to that in seen in the United States (35.8%, 1999–2002) [22].

In our study, 76% of hypertensive patients took antihypertensive medication and 63.9% of patients on antihypertensive medication had controlled HTN (Table 1). These results were high compared to those found by the Korean National Health and Nutrition Examination Survey (2005) [19], but this may be explained by the fact that our study population was derived from a highly selected group of patients who were visiting cardiology clinics for suspected or established cardiac diseases.

This study has some limitations. First, our study population was derived from a highly selected group of patients who were visiting tertiary hospitals for suspected or established cardiac diseases. Although it is appropriate to do a collective study with the cooperation of community based hospitals, public health centers, and general hospitals, our study included only tertiary hospitals. Therefore, cardiovascular risk factors are overrepresented in our sample. Second, this study was a simple cross-sectional study and did not consider information about practical stroke events. Therefore, we could not compare 10-year probability of stroke with real stroke incidence. Third, we did not directly compare the 10-year probability of stroke for our hypertensive sample and the KCPS cohort [10]. Fourth, we assessed smoking status with a single, self-reported questionnaire. Therefore, non-differential misclassification is possible.

## Conclusion

In conclusion, the average 10-year probability of stroke in our hypertensive sample was 26.27%, and this level was approximately 2.4 times higher than that seen in the KCPS cohort. The probability of stroke in our sample increased with severity of blood pressure in treated hypertensive patients. We suggest that stricter control of HTN may prove to be an important intervention for preventing stroke.

## Competing interests

The authors declare that they have no competing interests.

## Authors' contributions

Cheol Ung Choi participated in the design of the study, analysed and interpreted data and drafted the manuscript. Chang Gyu Park conceived of the study and participated in its design and critically revised the manuscript. All authors read and approved the final manuscript.

## Pre-publication history

The pre-publication history for this paper can be accessed here:


